# Issues on Trainability

**DOI:** 10.3389/fphys.2021.790196

**Published:** 2022-01-24

**Authors:** Zsolt Radak, Albert W. Taylor

**Affiliations:** ^1^Research Center for Molecular Exercise Science, University of Physical Education, Budapest, Hungary; ^2^Faculty of Sport Sciences, Waseda University, Tokorozawa, Japan; ^3^Faculty of Health Sciences, The University of Western Ontario, London, ON, Canada

**Keywords:** responders, non-responders, VO2max, resistance training, systemic adaptation

## Abstract

Trainability is an adaptive response to given exercise loads and must be localized to the targeted physiological function since exercise-induced acute and chronic adaptations are systemic. Lack of adaptation or moderate level of adaptation in one organ or one physiological function would not mean that other organs or functions would not benefit from exercise training. The most beneficial training load could easily be different for skeletal muscle, brain, the gastro-intestinal track, or the immune systems. Hence, the term of non-responders should be used with caution and just referred to a given organ, cell type, molecular signaling, or function. The present paper aims to highlight some, certainly not all, issues on trainability especially related to muscle and cardiovascular system. The specificity of trainability and the systemic nature of exercise-induced adaptation are discussed, and the paper aims to provide suggestions on how to improve performance when faced with non-responders.

## Introduction

Trainability is an important issue not only in elite sport but also for recreational athletes. It is often observed that a repeated exercise load does not cause increased performance for some subjects and those are referred to as non-responders (Bouchard and Rankinen, 2001; McLaren et al., 2018; Hortobagyi et al., 2021; Hrubeniuk et al., 2021; Mattioni Maturana et al., 2021). It is known that exercise-induced adaptation is different from the general adaptation theory developed by Selye (1975) since exercise-induced adaptation is specific. This specificity guarantees the necessity of different types and loads of training in various sports and explains the different phenotypes of sprinters, long-distance runners, or body builders.

One can suggest that non-responders are probably non-responders to a given training load and they can be responders when a personalized training load is given. Let’s take an example of those who are referred to as non-responders to improve maximal oxygen uptake (VO2max) by endurance exercise with moderate intensity (about 70% of VO2max). VO2max is referred to as a cardiovascular fitness measure, but VO2max is dependent on heart and arteriovenous oxygen difference (a-v difference) and can be measured by incremental exercise protocols in the laboratory or can be apprised by a huge number of field activities (Radák, 2018b). VO2max in very well trained athletes can reach 80 ml/kg/min. A-V difference is very much dependent on the capillarization and mitochondrial number of the skeletal muscle. The appropriate training to enhance cardiac output and to a-v difference could be easily different.

Most published work uses either moderate-intensity exercise, around 70% of the VO2max or high-intensity interval training (HIIT) to test the trainability of endurance. Moderate intensity training mostly results in general adaptation including enhanced cardiovascular function, while HIIT causes metabolism-induced adaptation to skeletal muscle (Radák, 2018a). It has been reported in a recent study that the adaptation to moderate-intensity training varied to a greater degree than after HIIT and non-responders were not found in the HIIT group (Maturana et al., 2021). Moreover, it would be interesting to test how non-responders adapt to mixed training, which contains both moderate and high-intensity training. In the present review, we aim to point out some important issues on trainability, in order to help understand exercise-induced systemic adaptation and we also offer some suggestions on how to improve performance, when faced with non-responders. In addition, it is important to note that exercise-induced adaptation is systemic, and non-responders in one function or organ could be responders in other functions and organs.

## Adaptation to a Single Bout of Exercise

Due to the nature of a single bout of exercise, the adaptive response is limited but it is systemic. One of the driving mechanisms that modulate adaptive response is the exercise-induced changes in metabolism and the generated metabolites. Since the degree of the elevation, the type of metabolism and the generated metabolites, and the main energy source of adenosine triphosphate (ATP) production are dependent upon the intensity of exercise, adaptation is dependent on intensity. In general, in the skeletal muscle, low-intensity exercise of long duration increases the resistance to fatigue, and high-intensity exercise leads to muscle strength and growth. At the cellular level, it can be measured by increased mRNA and short-lived protein levels. Exercise with a short duration and high intensity could elevate mRNA levels of enzymes involved in glycolytic metabolism because of the break-down of carbohydrate under anaerobic conditions, and the shortage of O2 delivery can activate hypoxia-inducible factor 1 (HIF1), and thus the inadequate availability of ATP can lead to activation of adenylate kinase, which can lead to phosphorylation of adenosine monophosphate-activated protein kinase (AMPK) and dependent cellular signaling and so forth. The decreased capacity to maintain the Na-K pump at the sarcolemma, the accumulation of metabolites such as lactate, ammonia, and inorganic phosphate, and the drop in creatine phosphate (CrP) levels could cause not only fatigue in the skeletal muscle, but they are also important initiators of adaptive response. This is very much true for reactive oxygen and nitrogen species (RONS) as well, which are produced at significant levels during high-intensity exercise (Radak et al., 2008a,^2011^, ^2013^). Single bouts of exercise with low and moderate intensity and long duration would cause increases in the mRNA expression of proteins involved in aerobic metabolism of sugars and fatty acids. Single bouts of exercise with low intensity can readily cause dehydration and increased body temperature. Blood glucose and muscle glycogen content could be significantly decreased. There are overlapping signaling pathways of high and low-intensity exercise bouts on various physiological processes, such as improving insulin sensitivity, regulation of mitochondrial network, etc. The release of microRNA-s from skeletal muscle to the circulation is also dependent on the intensity of exercise. Hence, resulting in different adaptive responses (Ramos et al., 2018; Torma et al., 2020).

It is very important to understand that exercise-induced adaptation is systemic. Lack of adaptation or moderate level of adaptation in one organ or one physiological function would not mean that other organs or functions would not benefit from exercise training. The most beneficial training load could be easily different for skeletal muscle, brain, the gastro-intestinal track, or the immune system. Hence, the term of non-responders should be used with caution and just referred to a given organ, cell type, molecular signaling, or function.

Physical exercise impacts the immune system via complex regulations, which involve proper adjustment of pro- and anti-inflammatory cytokine, and neopterin production (Scheffer and Latini, 2020). It is suggested that exercise-induced elevation of proinflammatory interleukin 6 (IL-6) even can block tumor necrosis factor alpha and attenuate IL-1β signaling, hence exhibiting anti-inflammatory effects (Pedersen, 2017). It also has been shown that exercise-induced modulation of metabolism alters proinflammatory responses in macrophages and the modulation can involve the energy sensor, AMPK (Nieman and Pence, 2020). Sirtuin 1 (SIRT1), the activity of which and its contents readily respond to exercise training (Radak et al., 2020) also promotes anti-inflammatory and tolerance programs in multiple immune cell types (Yoshizaki et al., 2010). Metabolites, which are the product of catabolic processes of proteins, carbohydrates, and fats are involved in immune defense and acute phase responses, complement activation, and humoral responses mediated by circulating immunoglobulins (Nieman and Pence, 2020). It is suggested that following a single bout of exercise, the immune system efficiency decreases, which provides an open window, which increases the chance of upper respiratory diseases (Kakanis et al., 2010). High-intensity exercise appears to increase the risk of upper respiratory track-related infections (Wang et al., 2012) and extreme exercise loads can also lead to the temporary weekend immune system (Simpson et al., 2020).

Indeed, the immune response is intensity dependent, since a single bout of high-intensity exercise is associated with a greater acute phase leukocyte count and redox response than aerobic exercise (Jamurtas et al., 2018). However, T lymphocyte and monocyte are important parts of the immune system, due to their angiogenic potential they are also important contributors of adaptive response by initiating to blood vessel growth and repair. It has been shown that a single bout of high-intensity exercise results in greater elevation of T lymphocyte and angiogenic Tie2-expressing monocytes than low-intensity acute exercise (O’Carroll et al., 2019).

Hormonal secretions can be changed by a single bout of exercise (Kraemer et al., 2020; Luse et al., 2020), which, due to the short time period, might not cause a long term adaptive response, but could be important to sharpen the sensitivity of receptors and initiate signaling processes. It has been shown that high-intensity exercise can increase the level of circulating anabolic hormones to a greater degree than high volume training (Wahl et al., 2013). Moreover, the effects of exercise on insulin sensitivity are also well described (DiMenna and Arad, 2021). A single bout of exercise, due to the altered regulation of blood supply, could readily cause ischemia in the liver, kidney, and gastro-intestinal track and affect the bacterial flora of the microbiome (Radak et al., 2019). Moreover, a single bout of exercise increases the levels of circulating microRNA (miR) (Denham and Prestes, 2016). These skeletal muscle-originated myo-miRs play a significant role in acute exercise-associated miR elevation. MiR(s) also provide a further control of translation, since they can readily lead to degradation of targeted mRNA (s) and thus prevent protein synthesis. Due to the systemic effects of exercise, oxygen and energy supply of the brain can be altered, which could cause modulation of neurotrophins, lactate uptake, etc. (Radak et al., 2019). Acute exercise can lead to increases in the level of circulating brain-derived neurotrophic factor (BDNF), and the results of animal studies have revealed that BDNF is increased following a single bout of exercise in the hippocampus of rats (Shahandeh et al., 2013).

When resistance exercise is done, acute resistance exercise with high muscle tension can cause damage to sarcomeres, which might be important in satellite cell proliferation, that can later on lead to myonuclear accretion (Damas et al., 2018). Moreover, high-intensity single exercise bouts activate phosphorylate transcription factors that, after repeated stimulation (which happens during chronic exercise), leads to increased muscle mass. When mouse skeletal muscle was treated with 50 high-intensity eccentric exercise, it turned out that serum response factor (SRF) activity is linked to a histone modification cascade starting with the phosphorylation of serine 10 on histone 3 (H3S10ph) (Solagna et al., 2020). The phosphorylation of histone can lead to increased protein synthesis which requires mitogen- and stress-activated kinase 1 (Solagna et al., 2020). The acute response of high-intensity exercise also causes phosphorylation of myocardin−related transcription factors on Ser66 (Solagna et al., 2020). The reversible modification of histone by acute exercise indicates that repeated exercise causes epigenetic modifications. Indeed, the effects of acute straight line running and running with 180-degree change of direction (mimicking ball game running) were studied on the DNA methylation of skeletal muscle and results revealed that overlapping methylation of many genes, and exercise specific methylation were also observed (Maasar et al., 2021). Acute exercise results decreased methylation of the whole genome, which could be an important step to initiate exercise-induced gene activation (Barres et al., 2012). It is important to note that the degree of the methylation of peroxisome proliferator-activated receptor gamma, coactivator 1 α (PGC-1 α), pyruvate dehydrogenase kinase, isoenzyme 4 (PDK4), and peroxisome proliferator-activated receptor δ (PPAR-δ), promoter regions were dependent on the intensity of a single bout of exercise (Barres et al., 2012), which further supports the idea that exercise-induced adaptation is dependent on the intensity of the exercise. The methylation of other organs is also modified by a single bout of exercise. It has been shown that acute restraint stress decreases global DNA methylation in the hippocampus, cortex, and periaqueductal gray in brain of rats, and this alteration was attenuated by a single bout of exercise, suggesting that exercise has the potential to modulate changes in DNA methylation and gene expression (Rodrigues et al., 2015).

**FIGURE 1:**
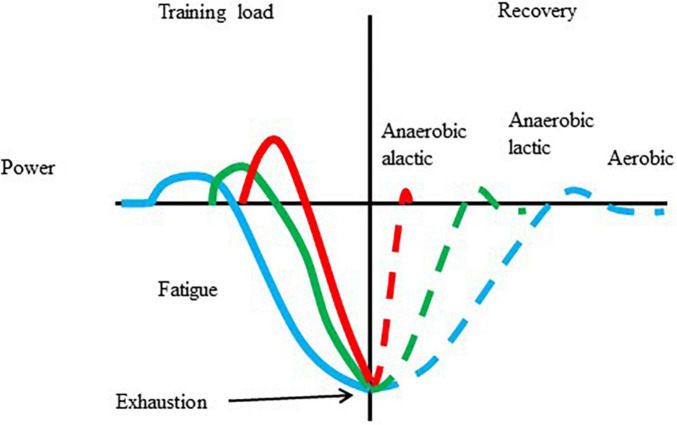
Adaptation to a single bout of exercise. Exercise results in increased metabolism, and power, which, after repeats can lead to fatigue and exhaustion. The length of recovery is dependent on the type of exercise that caused the fatigue and the level of fatigue. During the recovery periods overcompensation is possible.

The cellular and systemic responses to exercise with different intensities and durations are different. One of the well-known concepts of exercise-induced adaptation is shown in Figure 1. There is no reason to believe that a single bout of exercise, with over a certain intensity and duration, does not cause some of the biochemical, and physiological changes, which are the initiative of long-term adaptive responses. However, there is no guarantee that chronic training is always associated with enhanced performance.

## Adaptation to Long Term Exercise

The adaptation to long-term exercise is much more complex than the biochemical, and physiological responses to a single bout of exercise. Depending on the level of physical fitness, 4–12 weeks of training, 3–20 training sessions a week, with 30 to 180 min duration, could be necessary to improve sport performance. In order to increase the performance from a high level of physical fitness, one has to work very hard. Even daily 4–8 h of training is not exceptional for certain sports. This period of repeated training sessions with rest periods can lead to the synthesis of training intensity/duration-dependent targeted proteins and related improvement of targeted biochemical and physiological processes, including metabolism, receptor sensitivity, regulation of autonomic nervous system, and so forth.

Overall, the targeted reversible changes in histone and DNA modifications, the activation of signaling pathways, microRNA, and mRNA expressions that are observed as a result of a single bout of exercise, turn into production of proteins, alteration of neuro-endocrine regulation, and the immune system leads to improved physiological function and altered phenotype following repeated cyclic training loads (Denham et al., 2014; Howlett and McGee, 2016; da Silva et al., 2017; Quan et al., 2020). Regular exercise is carried out to bring about functional changes although in a different manner in most of the organs.

**FIGURE 2:**
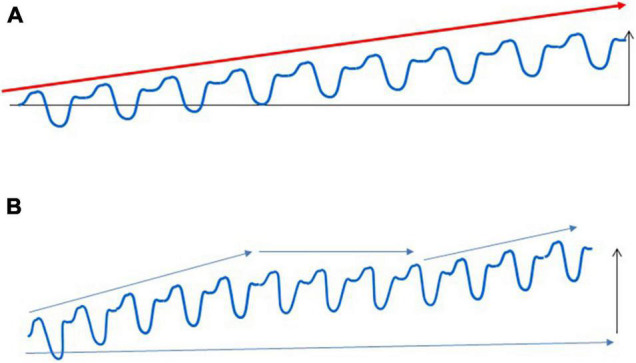
Adaptation to regular exercise bouts. Regular exercise does not cause a linear improvement in sport performance, which is shown in **(A)**. Long-term exercise training generally leads to increased performance for the untrained, but the adaptive response of trained subjects contains many periods with unchanged, sometimes even decreased performance **(B)**. These adaptive plateaus are a normal part of the adaptive response, and athletes whose preparation contains these plateaus cannot be regarded as non-responders.

On the other hand, it is also clear that long-term adaptation is not a linear process. Even with the best personalized training loads, there are periods with the absence of increased performance, which can last for weeks, months, or even for years (Figure 2). Plateaus are a normal part of the adaptive response, and the presence of a plateau does not mean that athletes are non-responders.

Among other influencing factors, adaptation is mainly dependent on the appropriate loading and resting cycles. It is generally accepted, that the improvement of those performance influencing factors is most meaningful, and the most significantly limiting factor for the performance. If we take an example of the athlete with an 8 L/min cardiac output, and 70% type I fibers in the significantly working skeletal muscle groups of his/her event, and whose training plan contains only moderate-intensity exercise loads, he/she might easily plateau during preparation, while this kind of training could benefit the same size of athletes with 6 L/min cardiac output. Sprint interval training (SIT) sessions or HIIT, on the other hand, could be quite useful to the 8 L mentioned athlete above, since these training sessions have very significant effects on muscle metabolism. It is also known that, due to the different recruiting thresholds of type I and type IIB and IIx fibers, the mitochondrial mass is increased by intensity and duration-dependent manner (Bishop et al., 2014). Therefore, if the given training intensity/duration is not tailored to the person it is easy to accept that the training does not cause measurable increase in the performance, and we can call those individuals non-responders. Given the enormous resources available to sport science it is not very difficult to design individual tailored training programs, to minimize the duration of plateaus and avoid non-responding training periods. It is important to note that exercise-induced adaptation is systemic, yet the question arises whether non-responders are non-responders in a systemic sense or simply in some physiological function.

## Trainability

It has been known for a long time that the same training program can result in different adaptive responses to different individuals and this observation was the basis for the development of personalized training loads. Interestingly, the training load which is used in most of the reported training studies is the same for all subjects. This can possibly explain the great variability of different training responses. Each subject participating in these studies could have a very different training experience, related to his/her inherited genetic setting, with different limits that influence training responses to the given load. As explained earlier, the ratio of fiber types could significantly influence the increase of mitochondrial mass to a given endurance training program, and the mitochondrial mass could influence VO2max, which often is used as a measure of trainability in endurance events. An animal model was recently introduced to study trainability (Koch et al., 2013). This model was set up using a genetically heterogeneous rat population (N/NIH stock) to develop lines named low response trainers (LRT) and high response trainers (HRT). Selection was based on the change in maximal running distance evaluated by a treadmill-running test to exhaustion. In the untrained condition, LRT and HRT rats were similar for exercise capacity. However, after receiving 9 weeks of a standard amount of endurance training, HRT rats improved, on average, by 200 meters for distance run whereas those bred as LRT failed to improve and, on average, declined in running capacity by 65 meters (Koch et al., 2013). We tested the different adaptive response of LRT and HRT rats after 3 month of endurance training at 70% of VO2max (Marton et al., 2015). We found that the alterations in the levels of VO2max, RONS, SIRT1, NAD (+)/NADH ratio, proteasome (R2 subunit), and mitochondrial network-related proteins such as mitochondrial fission protein 1 (Fis1) and mitochondrial fusion protein (Mfn1) were not related to trainability for these rats. However, data suggested that PGC1-α, nuclear respiratory factor 1 (NRF1), mitochondrial transcription factor A (TFAM), and Lon protease might be linked to trainability to the given exercise protocol. These results further suggest that HIIT training could possibly be more effective to train the LRT group, since HIIT more readily induced PGC1-α, NRF1, TFAM than aerobic exercise (Williams et al., 2019). The lack of non-personalized training, therefore, may be one of the reasons for the lack of a training response.

Nutritional habits could directly impact trainability. One interesting example is the possible effect of antioxidant supplementation on performance. The general belief is that antioxidants cannot directly improve exercise performance but could play an important role in preventing or attenuating exercise-induced muscle damage (Sureda et al., 2014; Decroix et al., 2018; Nocella et al., 2019). However, research-based opinions suggest pro and contra roles on the effects of antioxidant supplementation on adaptive responses. RONS are important signaling molecules of exercise-induced adaptation (Davies et al., 1982; Radak et al., 2008b; Torma et al., 2020), but is it possible that Vitamin C and E cocktails eliminate the systemic effects of exercise? This is certainly not the case. Antioxidant supplementation could down-regulate some cellular signaling processes (Gomez-Cabrera et al., 2008) and it has been shown that although the VO2max of the subjects increased from 41.2 to 45.6 ml/kg/min after 9 weeks exercise with daily 1 g of Vitamin C supplementation the increases were not significant, while untreated groups showed significant increases (Gomez-Cabrera et al., 2008). Therefore, the Vitamin C supplementation attenuated the effects of exercise, in a given group, but this did not eliminate or curb the beneficial effects. Indeed, it seems unlikely that the complex effects of exercise can be blocked by antioxidant supplementation (Higashida et al., 2011). The beneficial effect or the possible attenuation of exercise-induced adaptation of antioxidant supplementation could be dependent on the timing of supplementation and the level of physical fitness (Radak et al., 2017). A great number of elite athletes use antioxidant supplementation in order to support exercise performance. However, it seems improbable that antioxidant supplementation would increase or decrease exercise performance to a measurable degree (Reid, 2016; Bowtell and Kelly, 2019; Higgins et al., 2020; Arazi and Eghbali, 2021). According to our present knowledge, it seems unlikely that the antioxidant supplementation could create a non-responding group to exercise training.

What about trainability and resistance training? It is clear from the literature that protein uptake can directly influence the rate of muscle metabolism and, up to a degree, the development of muscle hypertrophy (Morton et al., 2018a). However, the intensity and duration of resistance training are the main factors of exercise-induced muscle plasticity. Results suggest that sensitivity of tension-mediated signaling pathways and the number of androgen receptors could make the difference between responders and non-responders (Morton et al., 2018b). Moreover, it has also been suggested that the difference in biogenesis of ribosomes (Figueiredo et al., 2015) and satellite cell proliferation capacity (Petrella et al., 2008) could be factors. Knocking out the paired box 7 (Pax7) gene, which is often used to identify satellite cells, results in muscle weakness and early death (Kuang et al., 2006). The difference in the expression patterns of miR (s) (Davidsen et al., 2011; Ogasawara et al., 2016) could also account for the different training responses to resistance training. All of these training adaptation limiting factors could be the result of a different genetic setting, in other words, the adaptation to training is very individual including epigenetic modifications that are due to exercise training, nutritional habits, and other lifestyle and environmental factors (Voisin et al., 2015).

One of the exercise-dependent controlling factors of adaptation is the methylation of DNA and post-translational modification of histone residues. Hypo-and hyper-methylation of CpG island of the promoter regions of genes can activate and silence transcription and directly alter adaptive responses. A single bout of exercise results in hypomethylation of whole DNA, and the promoter region of some genes which are important in exercise-induced adaptation (Barres et al., 2012), suggesting a regulatory role of methylation in trainability. Due to modulating role of exercise on methylation, the plasticity of this system makes it possible to convert non-responders to responders and vice versa. The adaptability or trainability to a given exercise program is initiated by methylation-controlled transcription, followed by translation. This notion is supported by the observation that the short-chain fatty acid, butyrate, which is produced at an elevated rate by the gut microbiome is an adaptive response to exercise training (Abraham et al., 2019) can readily change DNA methylation in fibroblasts (Parker et al., 1986). Although the direct evidence that methylation readily impacts trainability is rare, the health-associated consequences of exercise modulated DNA methylation are well known. It turns out that regular exercise hypermethylates the TRIM59 gene, which is a powerful oncogene, and KLF14 genes, which regulates inflammation (Spolnicka et al., 2018). The downregulation of these genes is associated with the anti-tumor and anti-inflammatory activities of regular exercise (Spolnicka et al., 2018).

**FIGURE 3:**
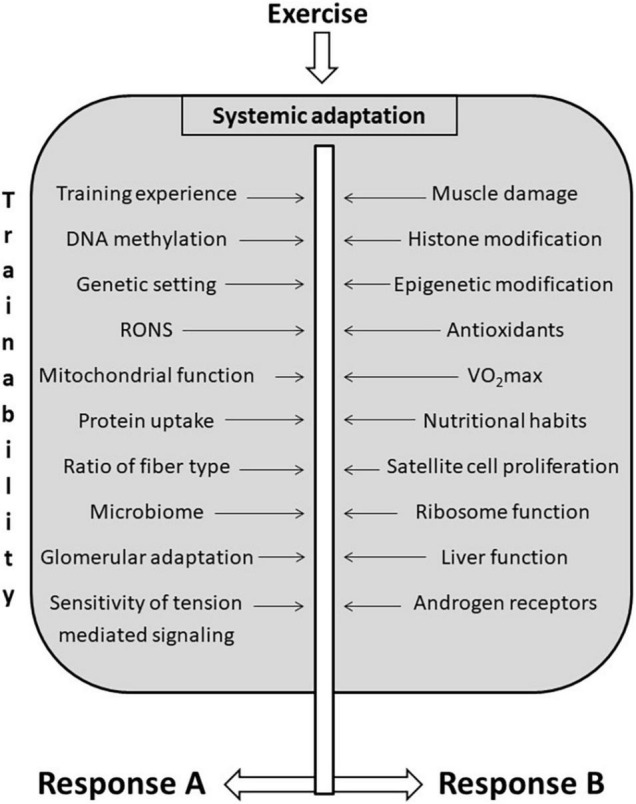
Factors that influencing trainability. There are a great number of factors that influence trainability of different organs. Exercise-induced adaptation is systemic, and the same training load could cause different adaptive responses in different organs indicated as **(A,B)**, to different functions, and to different individuals.

Trainability is a complex phenomenon because exercise-induced adaptation is systemic (Figure 3). Most trainability studies focus on the adaptive response to the targeted training. However, adaptation in organs distant from skeletal muscle could significantly affect sport performance and health. The exercise-induced adaptation of liver directly affects exercise performance because liver controls lactate (Proia et al., 2016), carbohydrate, fat, and protein metabolism, partly by the generation of hepatokines such as Fibroblast Growth Factor 21, Fetuin-A, Angiopoietin-like protein 4, and Follistatin (Ennequin et al., 2019). Glomerular adaptation to exercise training, which involves filtration of lactate, Na^+^ and K^+^ and the adjustment of pH and hydration also directly alters exercise performance (Mallie et al., 2002). The adaptive response of training could lead to increased contractile response to sympathetic agonists of renal arteries as a result of a significantly reduced blood flow during exercise bouts (Kocer et al., 2011). Moreover, the systemic effects of adaptation to exercise training cover the adaptive response of the microbiome as well. It has been shown that endurance exercise resulted in elevation of the relative abundance of *Veillonella atypica* in the gut microbiome (Scheiman et al., 2019). This bacterium converts fatigue-inducing lactate into energy-providing propionate, and hence, supports endurance performance (Scheiman et al., 2019). At the moment we don’t have enough information on how the intensity of exercise training influences microbiome and the relative abundance of different bacterial strains. It is known that very long term exhaustive exercise can easily cause gut ischemia and gastrointestinal problems (de Oliveira and Burini, 2009), which most probably would differently alter the microbiome status of the gastrointestinal track. Using Zucker rats it was found that although HIIT was more effective to decrease epididymal fat mass than moderate-intensity exercise, the microbiome contents did not differ significantly (Maillard et al., 2019).

The repeated exercise bouts associated with energy demands result in adaptive responses to liver, increasing fat oxidation, which serves a protective role against fatty liver diseases (Shephard and Johnson, 2015). Regular exercise beneficially affects the function of kidneys by maintaining mitochondrial function and suppressing inflammation (Radak et al., 2020). Regular exercise can easily lead to increased brain-derived neurotrophic factor (BDNF) concentration in the brain, and elevated BDNF beneficially affects brain plasticity, memory, mood, and viability of neurons (Gomez-Pinilla and Hillman, 2013; Kujach et al., 2019; Quan et al., 2020).

The present paper intends to show the complexity of exercise-induced systemic adaptation and point out how difficult it could be to divide the subjects into responders and non-responders, when the whole body is responding. Despite the systemic adaptation, the organ-dependent adaptation varies a lot at different intensities and loads, and the so-called optimal training load is different for different organs. Overall, trainability is considered to be the adaptive response of the targeted condition. Nonetheless, it must be kept in mind that the exercise performance is dependent on the response of many organs and a large range of influencing factors. The lack of or attenuated improvement of, the targeted condition to exercise training, might have many causes including adaptive plateau, improper loading, genetic limitation, epigenetic alteration, and so forth.

## Limitation of the Study

One of the great limitations is the complexity of trainability, which appears to be very individual, and as of it, uniform approaches can provide limited information. Indeed, vast range of trainability studies used only one exercise protocol to all subjects without pointing out the individual limiting factor (s) of the targeted training goal. Therefore, the review of the results of these studies probably cannot provide realistic data on trainability. There is a huge limitation to gain functional results of heart, brain, liver, kidney, immune system, microbiome, and other organs especially in human studies, however, these elements can directly or indirectly affect trainability. The other limiting factor could be the possible plasticity of trainability, as we change one limiting factor the following limiting factor could be changed by very different training and the possible interactions are not well known. The present paper could just show a small part of trainability, which is part of an extremely complex adaptive response to exercise.

## Future Perspectives

Appropriate testing of physiological functions of different systems and organs, like muscle and cardiovascular system and brain, before and after different training loads would be important to better understanding exercise-induced adaptive response. Moreover, using biomarkers to assess liver-, kidney-, and immune function along with the status of the microbiome would also add a lot to understanding the enigma of trainability.

## Author Contributions

Both authors listed have made a substantial, direct, and intellectual contribution to the work, and approved it for publication.

## Conflict of Interest

The authors declare that the research was conducted in the absence of any commercial or financial relationships that could be construed as a potential conflict of interest.

## Publisher’s Note

All claims expressed in this article are solely those of the authors and do not necessarily represent those of their affiliated organizations, or those of the publisher, the editors and the reviewers. Any product that may be evaluated in this article, or claim that may be made by its manufacturer, is not guaranteed or endorsed by the publisher.
